# Supplementary material for: QTL mapping of human retina DNA methylation identifies 87 gene-epigenome interactions in age-related macular degeneration
Source: Nat Commun. 2024 Mar 4;15:1972. doi: 10.1038/s41467-024-46063-8 (PMC10912779; doi:10.1038/s41467-024-46063-8)
Supplement: Supplementary file 1 — Supplementary Information [file 41467_2024_46063_MOESM1_ESM.pdf]

QTL mapping of human retina DNA methylation identifies 87 gene-epigenome interactions in age-related macular degeneration

Jayshree Advani[1], Puja A. Mehta[2,3,4], Andrew R. Hamel[2,3,4], Sudeep Mehrotra[2,3,4], Christina Kiel[5], Tobias Strunz[5], Ximena Corso-Díaz[1], Madeline Kwicklis[1], Freekje van Asten[1], Rinki Ratnapriya[1], Emily Y. Chew[6], Dena G. Hernandez[7], Sandra R. Montezuma[8], Deborah A. Ferrington[8, #], Bernhard H. F. Weber[5,9], Ayellet V. Segrè[2,3,4, *], Anand Swaroop[1, *]

[1]Neurobiology, Neurodegeneration and Repair Laboratory, National Eye Institute, National Institutes of Health, Bethesda, Maryland, USA

[2]Ocular Genomics Institute, Department of Ophthalmology, Massachusetts Eye and Ear, Boston, Massachusetts, USA

[3]Department of Ophthalmology, Harvard Medical School, Boston, Massachusetts, USA

[4]Broad Institute of MIT and Harvard, Cambridge, Massachusetts, USA

[5]Institute of Human Genetics, University of Regensburg, Regensburg, Germany

[6]Division of Epidemiology and Clinical Applications, Clinical Trials Branch, National Eye Institute, National Institutes of Health, Bethesda, MD, USA

[7]Laboratory of Neurogenetics, National Institute of Aging, National Institutes of Health, Bethesda, Maryland, USA

[8]Department of Ophthalmology and Visual Neurosciences, University of Minnesota, Minneapolis, Minnesota, USA

[9]Institute of Clinical Human Genetics, University Hospital Regensburg, Regensburg, Germany

[#]Present address: Doheny Eye Institute, Pasadena, California, USA

***Corresponding authors:**

Anand Swaroop, Ph.D., Neurobiology, Neurodegeneration & Repair Laboratory, National Eye Institute, National Institutes of Health, Bethesda, Maryland, USA. Phone: (301) 435-5754. Email: swaroopa@nei.nih.gov

Ayellet V. Segrè, Ph.D., Ocular Genomics Institute, Department of Ophthalmology, Massachusetts Eye and Ear, Harvard Medical School, Boston, Massachusetts, USA. Phone: 617-573-3025. Email: ayellet_segre@meei.harvard.edu

Contents

1. Supplementary Figures and Legends

Supplementary Figure 1

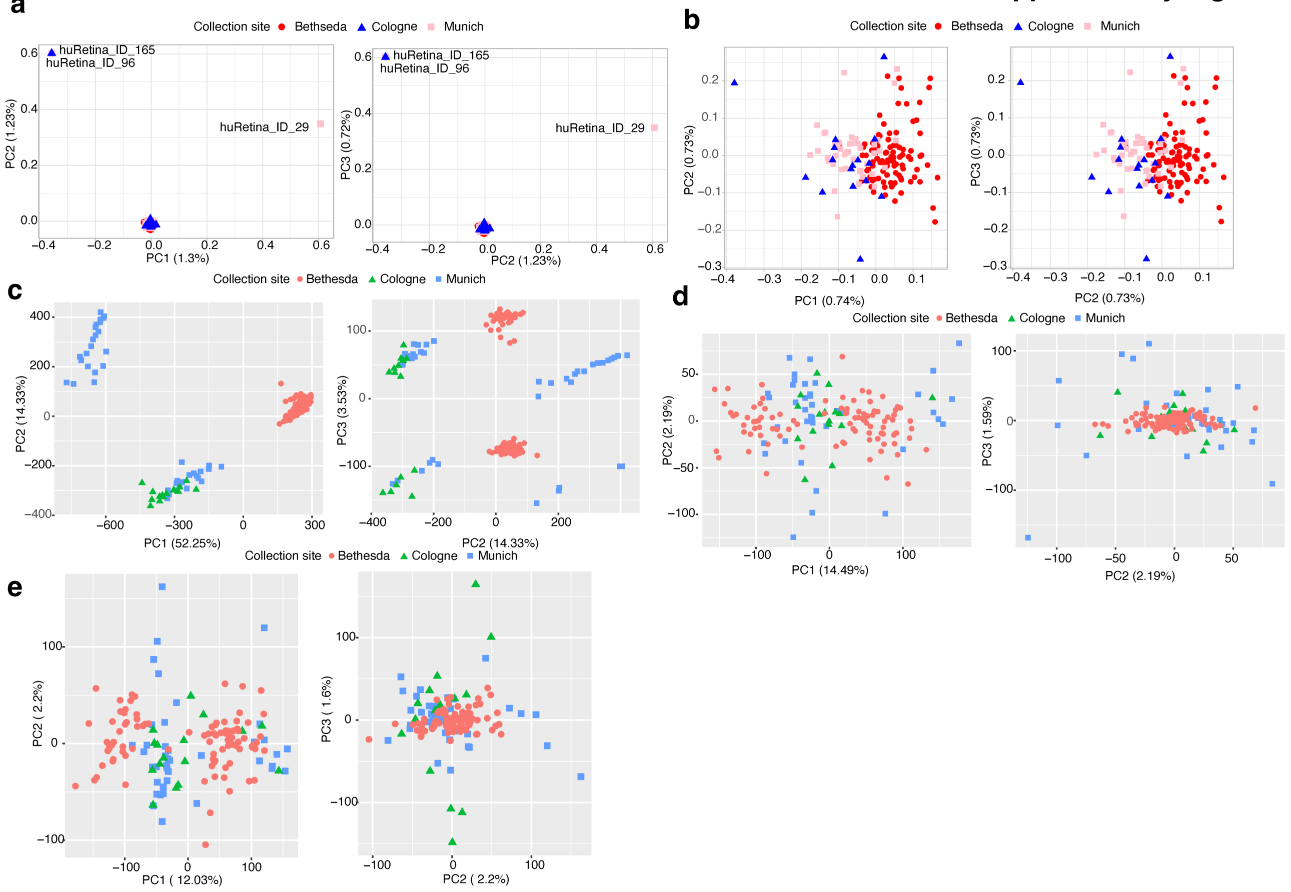

Supplementary Figure 1:

a: PCA plots of donors with sample collection site based on genotype data. b: PCA plot of donors with sample collection site after removal of outliers based on genotype data. c: PCA plots of donors with sample collection site based on normalized methylation levels. d: PCA plots of donors with sample collection site based on normalized methylation levels after batch correction with 38SVs. e: PCA plots of donors with sample collection site based on normalized methylation levels after batch correction with 15 SVs.

Supplementary Figure 2

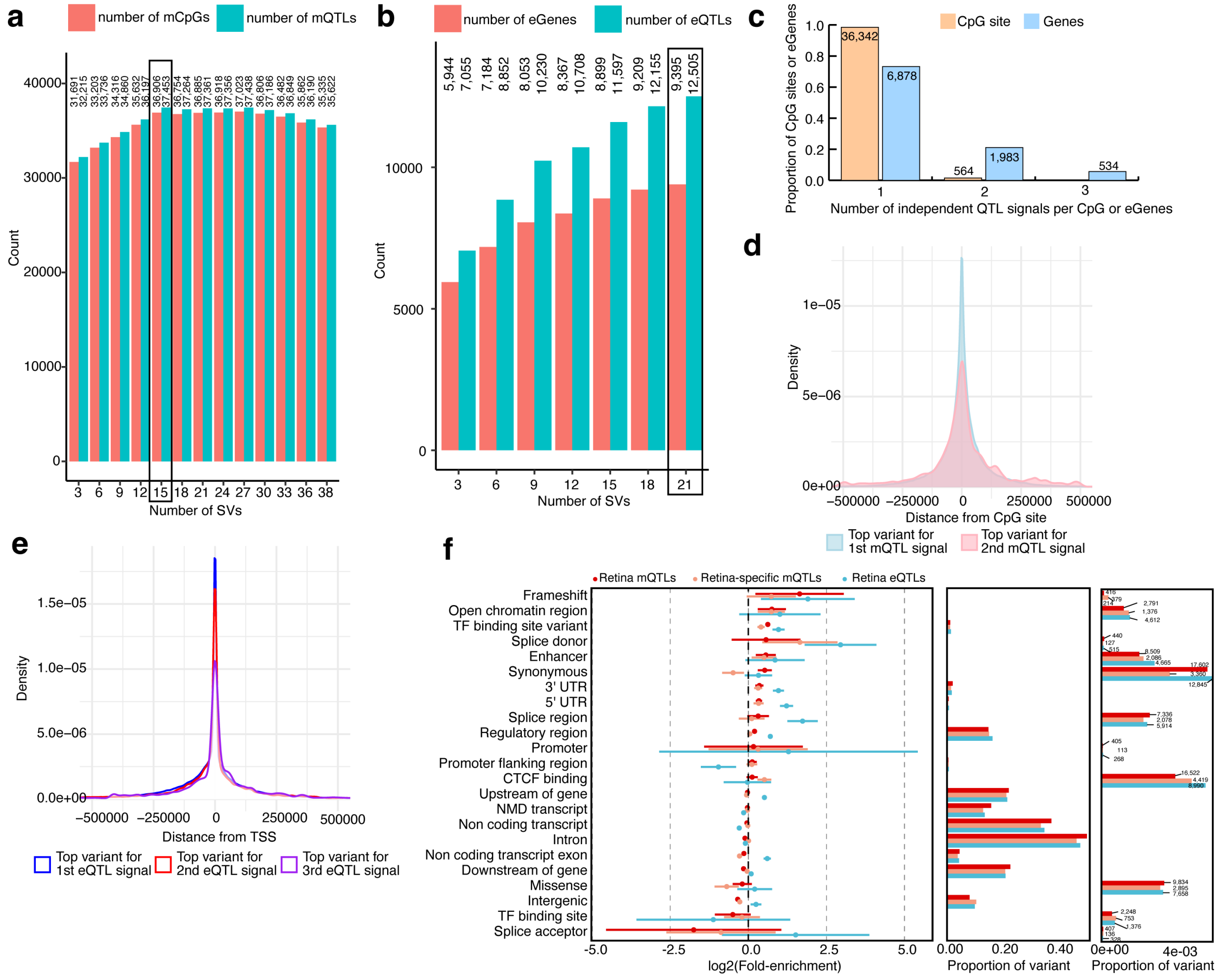

Supplementary Figure 2:

a: Distribution of number of mCpGs and mQTLs identified from n=152 biologically independent samples at different number of surrogate variables (SVs) b: Distribution of number of eGenes and eQTLs identified from n=403 biologically independent samples at different number of surrogate variables (SVs). c: The distribution of *cis*-independent mQTLs/eQTLs signals for CpG sites or eGenes respectively based on the conditional analysis in QTLtools. d: Comparing the distribution of distances of each *cis*-mQTL variant to its target CpG between the 1st and 2nd independent signals, zooming into a 500kb window e: Comparing the distribution of distances of each *cis*-eQTL variant to its target eGene's transcription start site (TSS) between the 1st, 2nd and 3rd independent

signals, zooming into a 500kb window. f: QTL enrichment in functional annotations for all retina (red) or retina-specific (orange) *cis*-mQTLs and all retina *cis*-eQTLs (blue). Points (centre) refer to m/eQTL fold-enrichment estimates on log2 scale with 95% confidence intervals (lines), shown in descending order based on the retina mQTL fold-enrichment across annotations with >550 variants per QTL type. All significant variant-gene pairs per target CpG or gene were analyzed with TORUS. Proportion of m/eQTL variants per annotation is shown in the histogram with the right-hand histogram zooming in for proportions below <=0.0071.

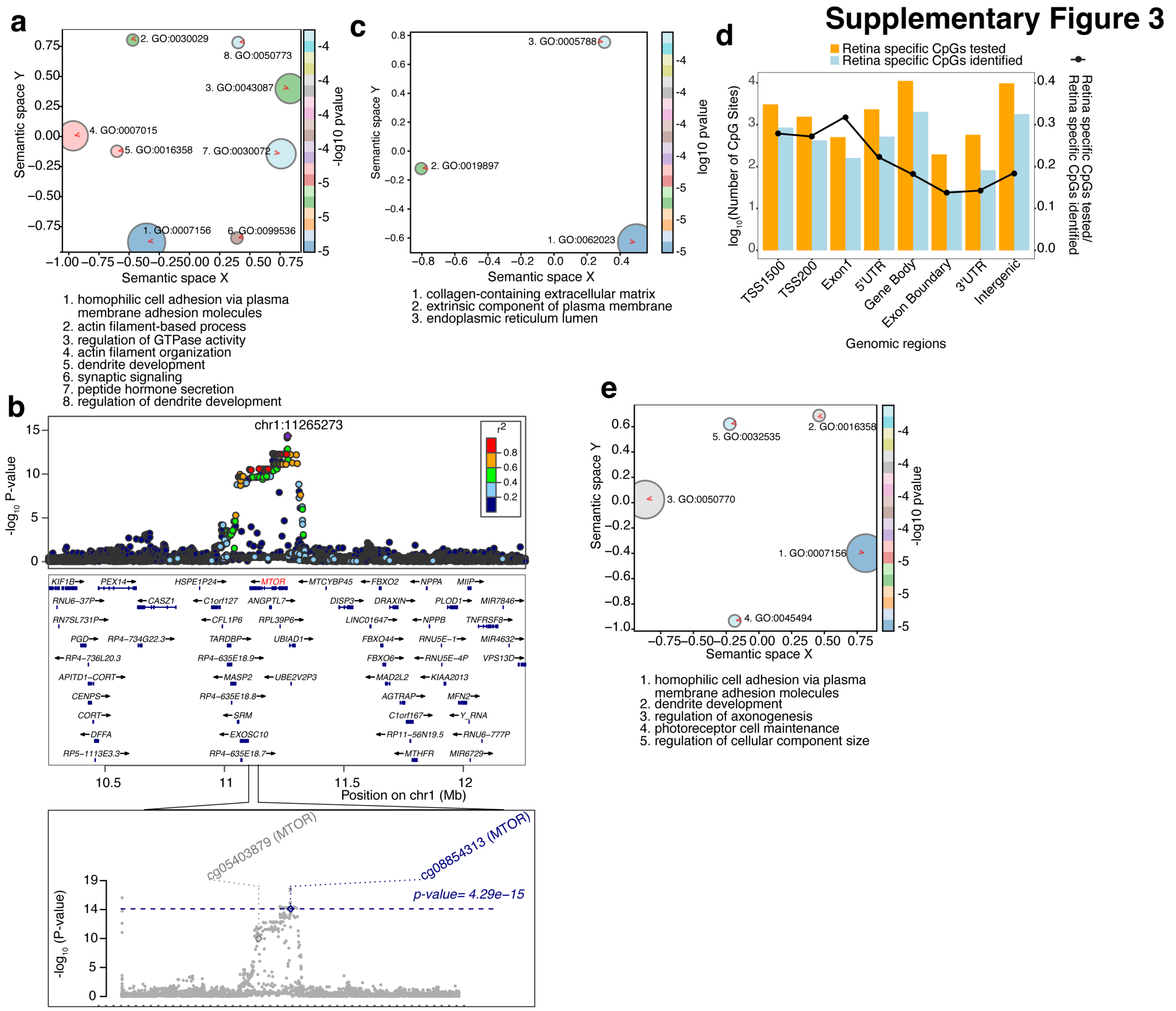

Supplementary Figure 3:

a: Enriched biological processes identified in the gene ontology analysis of top 10% of mGenes from retina mQTLs using GeneEnrich (adj. P<0.05). b: LocusZoom plot showing the retina mQTL associations, -log (*P* value) for cg08854313, the CpG with the top mQTL signal in the locus (P value = 4.29 x 10^{-15}). cg08854313 is located in the 1st exon of *MTOR* and the most significant mVariant, rs7517155 is located in the intronic region of *MTOR*. The diamond indicates the top mVariant (chr1:11265273:T:A; rs7517155) for the independent cg08854313 mQTL signal. The color of the points is determined by their linkage disequilibrium (LD) with respect to the lead mVariant in purple. The bottom plot shows $-\log_{10}$(P value) of the variant associations with two

different CpGs in the *MTOR* gene region from mQTL analysis. The grey and blue diamonds represent $-\log_{10}$(*P* value) of the lead mVariants for CpGs cg05403879 and cg08854313, respectively. c: Enriched biological processes identified in the gene ontology analysis of top 10% of eGenes from retina eQTLs using GeneEnrich (adj. P<0.05). d: Number of retina specific CpG sites tested (orange) and identified as significant (light blue) in various genomic regions in the mQTL analysis. e: Enriched biological processes identified in the gene ontology enrichment analysis of retina- specific CpGs using GeneEnrich (adj. *P*<0.05). Plots in panels a, c, e were generated for the non-redundant sets of significantly enriched gene ontology terms using GO-Figure v1.0.1.

Supplementary Figure 4

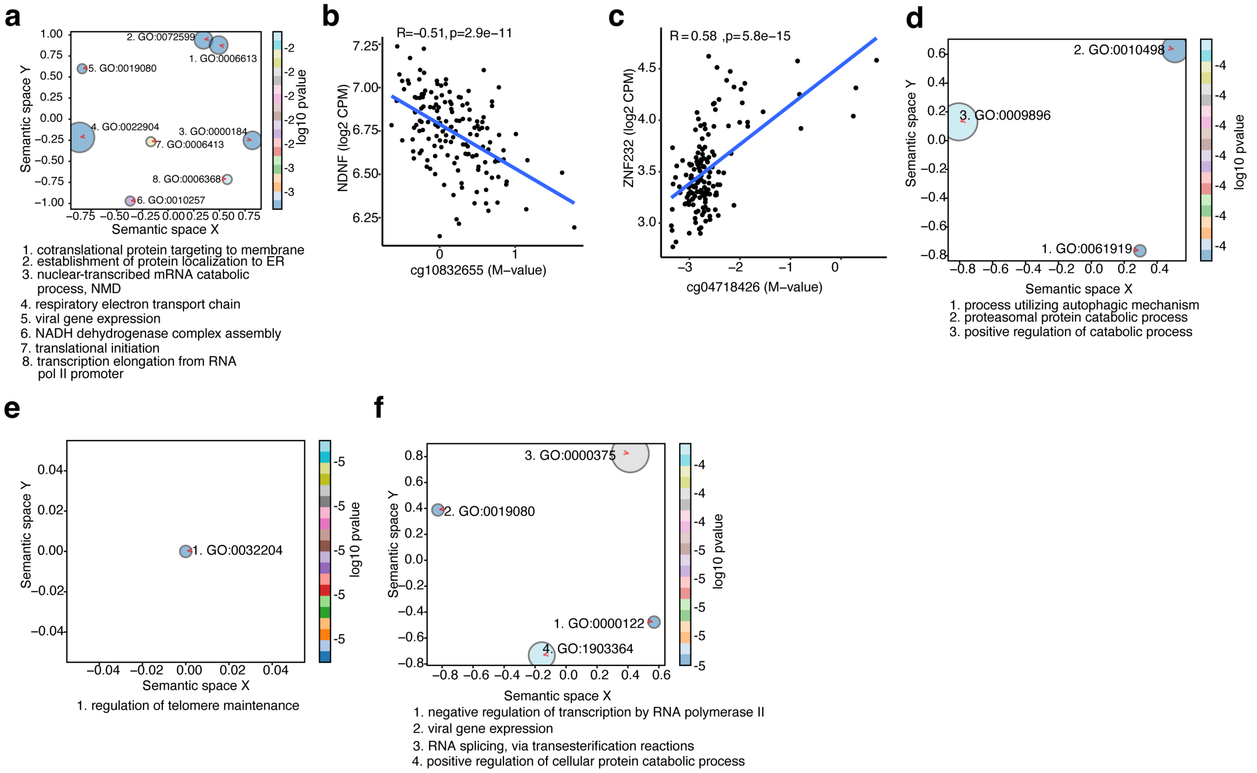

Supplementary Figure 4:

a: Enriched biological processes identified in the gene ontology analysis of top 10% of target genes of eQTMs. b: eQTM for CpG cg10832655 located in gene body and *NDNF* on chromosome 4 with R=-0.51, p=2.9 x 10^{-11}. c: eQTM for CpG cg04718426 located in gene body and *ZNF232* on chromosome 17 with R=0.58, p-=5.8 x 10^{-15}. d: Enriched biological processes identified in the gene ontology analysis of target genes which regulate more than 10 eQTMs. e: Enriched biological processes identified in the gene ontology analysis of target genes of chr16. f: Enriched biological processes identified in the gene ontology analysis of target genes of chr19. In panels a, d, e, f, gene ontology enrichment analysis was performed using GeneEnrich, and the non-redundant set of significantly enriched gene ontology terms (adj. P<0.05) were identified and plotted using GO-Figure v1.0.1.

Supplementary Figure 5

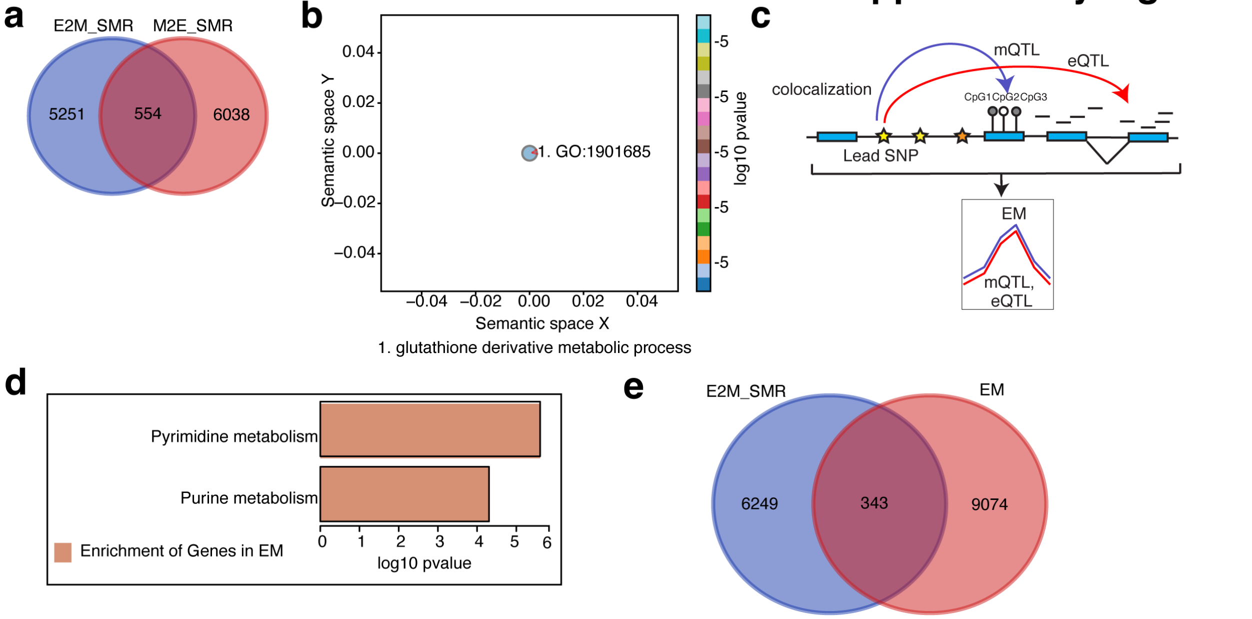

Supplementary Figure 5:

a: Venn-diagram representing common and unique associations identified in E2M_SMR and M2E_SMR analysis. b: Enriched biological processes identified in the gene ontology analysis of common genes of E2M_SMR and M2E_SMR using GeneEnrich (adj. P<0.05). Figure generated with GO-Figure v1.0.1. c: Schematic representation of colocalization analysis of eQTL and mQTL. d: Bar graph representing the enriched pathways of genes identified in EM of coloc, based on GeneEnrich (adj. P<0.05). e: Venn-diagram representing common and unique associations identified in E2M_SMR and EM of coloc.

Supplementary Figure 6

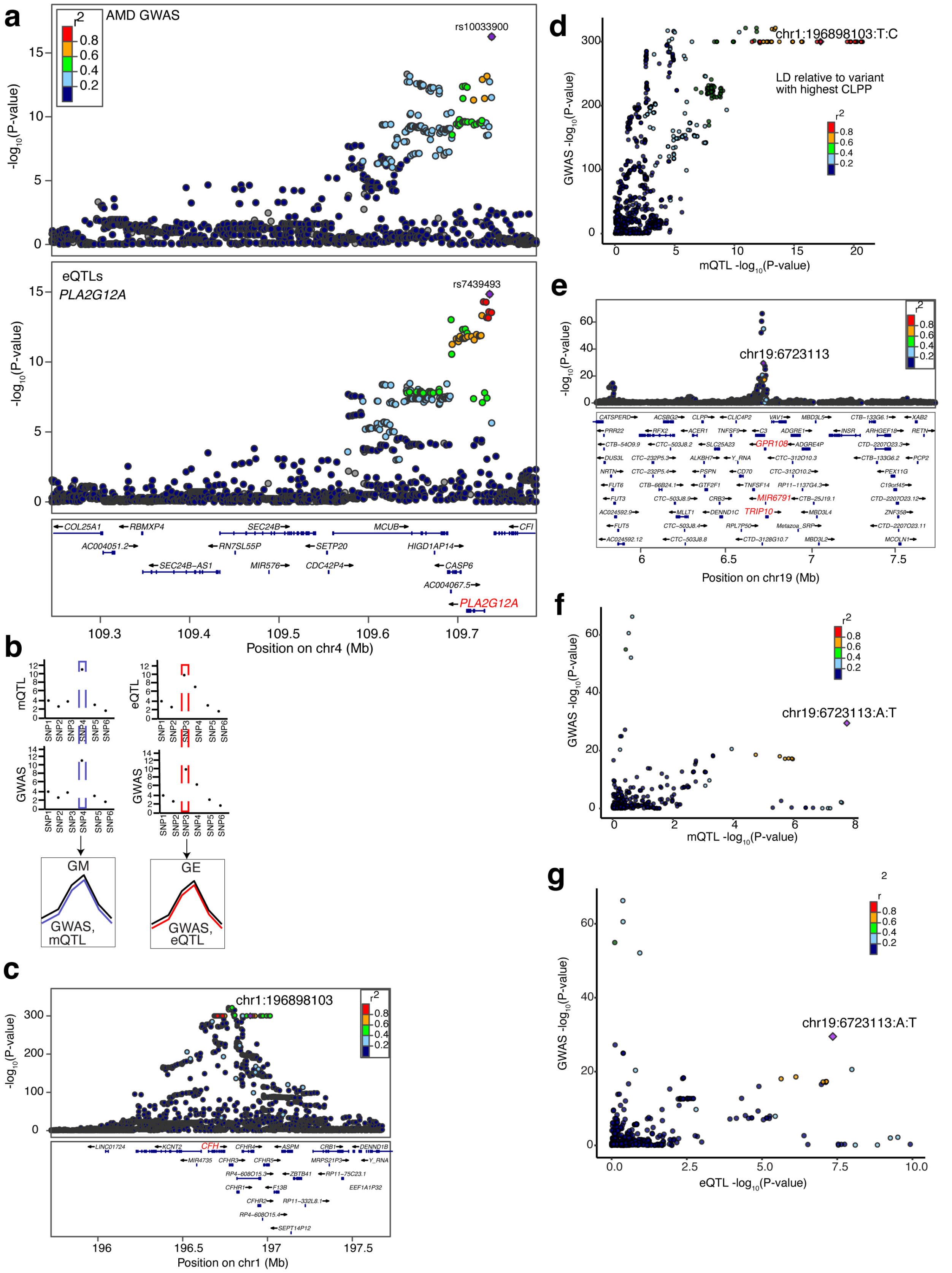

Supplementary Figure 6:

a: LocusZoom plots of GWAS (genotype and AMD GWAS association), *PLA2G12A* eQTLs (genotype and *PLA2G12A* expression association). The y axis shows $-\log_{10}$(P value) of association tests from GWAS and eQTLs. Highlighted variant rs10033900 is the lead GWAS variants in the locus, and variant rs7439493 is the most significant eVariant in SMR analysis of AMD GWAS and eQTLs. b: Schematic of representation of colocalization analysis with eCAVIAR aligns the causal variants of mQTL and AMD GWAS, and eQTL and AMD GWAS. c: LocusZoom plot between the AMD GWAS and the retina mQTL signals for cg04827773 (CFH). $-\log_{10}$(P value) of AMD GWAS with points color coded based on linkage disequilibrium (LD) (r^2) relative to cg04827773 (CFH), with highest colocalization posterior probability (CLPP). d: LocusCompare plot comparing $-\log_{10}$(P value) of AMD GWAS to $-\log_{10}$(P value) of retina mQTLs acting on cg04827773 (CFH). e: LocusZoom plot between the AMD GWAS and the retina mQTL signals for cg07567260 (*GPR108*/*TRIP10*/*MIR6791*). $-\log_{10}$(P value) of AMD GWAS with points color coded based on LD (r^2) relative to cg07567260 (*GPR108*/*TRIP10*/*MIR6791*), with highest colocalization posterior probability (CLPP). f: LocusCompare plot comparing $-\log_{10}$(P value) of AMD GWAS to $-\log_{10}$(P value) of retina mQTLs acting on cg07567260 (*GPR108*/*TRIP10*/*MIR6791*). g: LocusCompare plot comparing $-\log_{10}$(P value) of AMD GWAS to $-\log_{10}$(P value) of retina eQTLs acting on *GPR108* (*GPR108*/*TRIP10*/*MIR6791*). f, g: Points are color coded based on LD (r^2) relative to the variant with highest CLPP.

Supplementary Figure 7

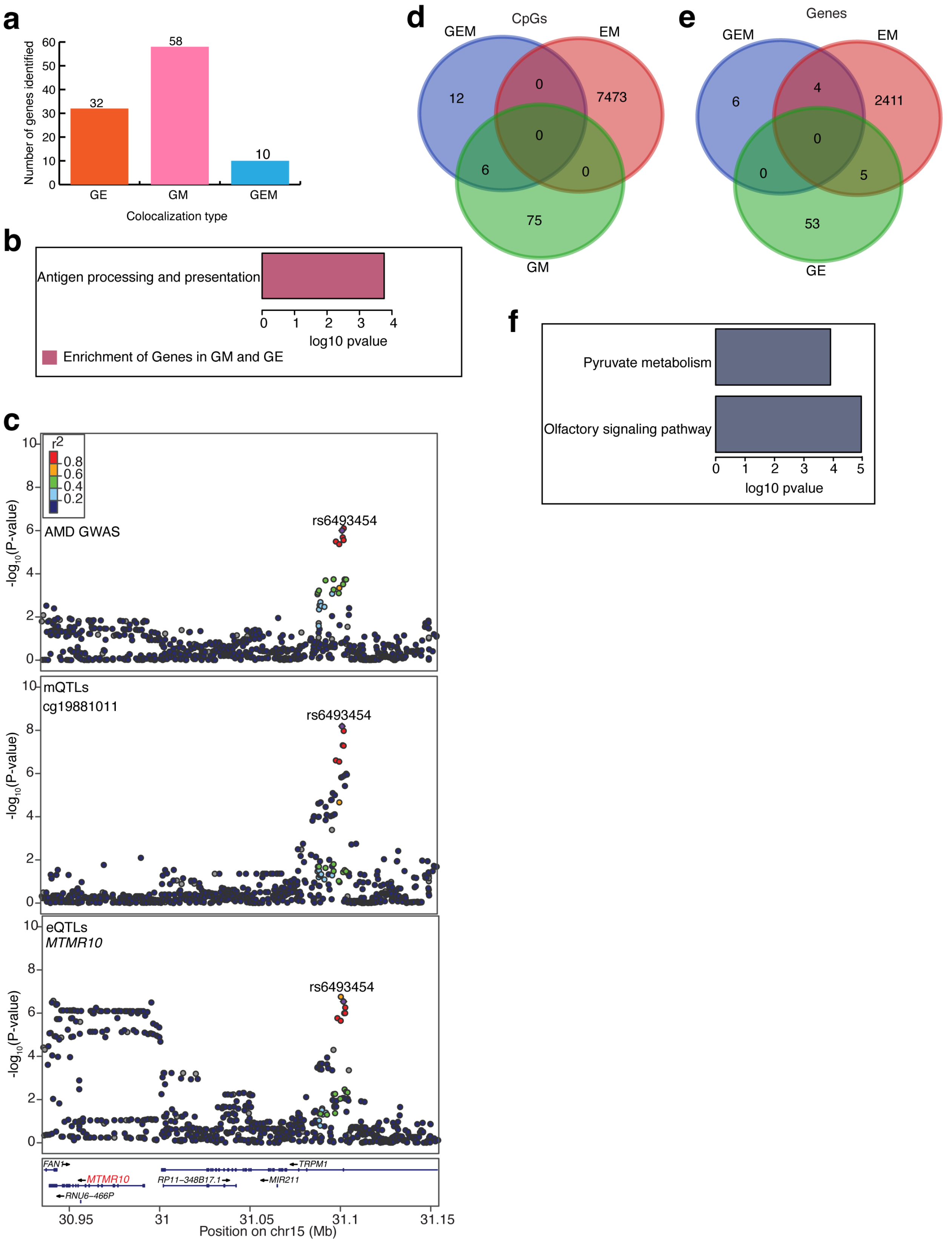

Supplementary Figure 7:

a: Number of genes identified in colocalization across AMD GWAS, mQTL and eQTL using coloc. b: Bar graph representing the enriched pathways of genes identified in GM and GE of coloc (GeneEnrich FDR < 0.05). c: LocusZoom plots of GWAS (genotype and AMD GWAS association), CpG cg21851553 mQTLs (genotype and cg21851553 methylation association), and *MTMR10* eQTLs (genotype and *MTMR10* expression association) in retina. The y axis shows $-\log_{10}$(P value) of association tests from GWAS, mQTLs and eQTLs. Points are color coded based on linkage disequilibrium (LD) (r^2) relative to the highlighted variant rs6493454, that was most significant in moloc analysis of AMD GWAS, mQTLs and eQTLs (GEM) in the locus. d: Venn-diagram representing common and unique retina CpGs significant in GEM, EM, and GM of coloc and moloc. e: Venn-diagram representing common and unique genes significant in GEM, EM, and GM of coloc and moloc. f: Bar graph representing the enriched pathways (GeneEnrich FDR < 0.05) of mGenes mapped to common CpGs identified in E2M_SMR, M2E_SMR and EM of coloc.

Supplementary Figure 8

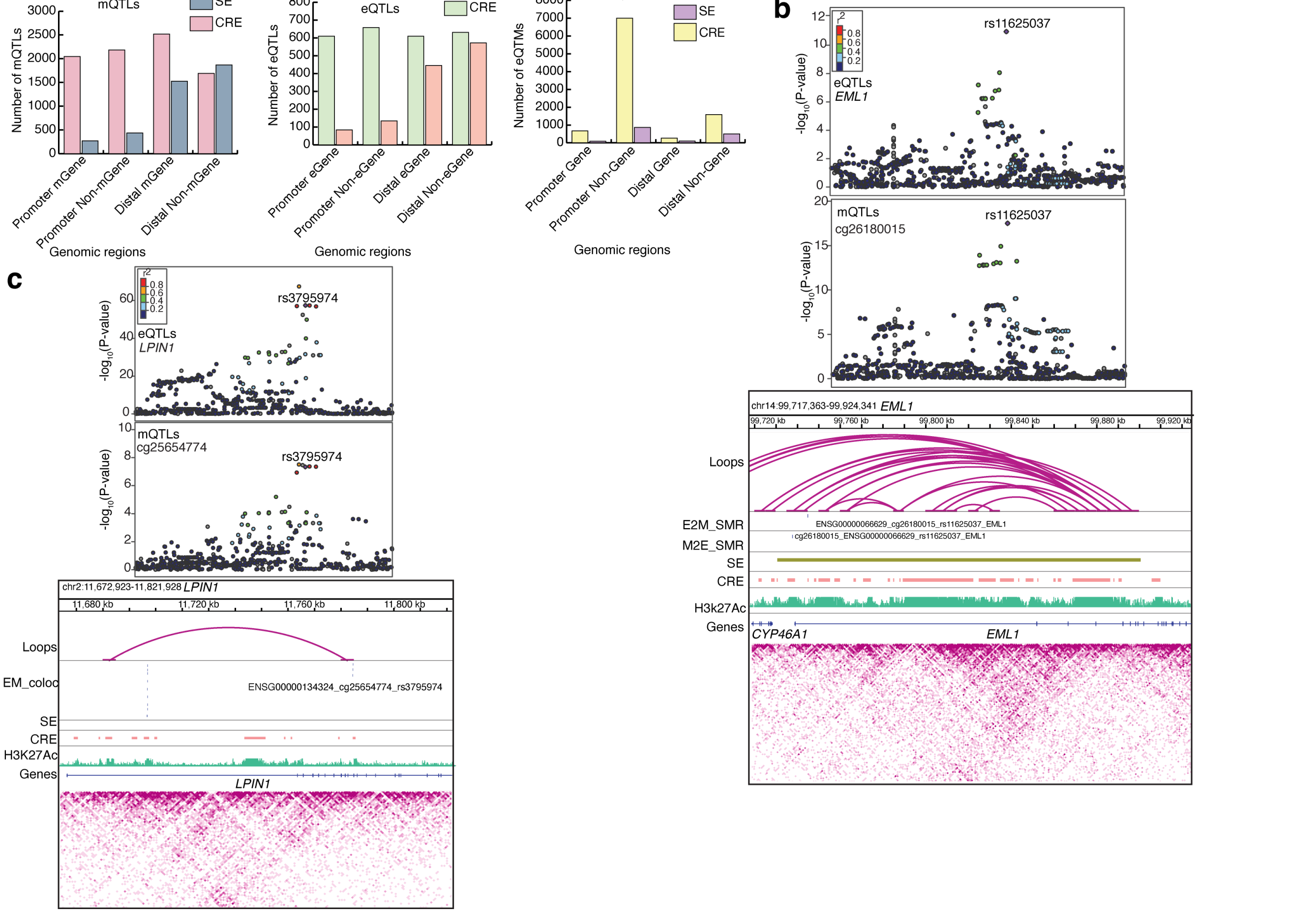

Supplementary Figure 8:

a: Number of unique retina mQTLs, eQTLs and eQTMs overlapping a retina CRE indicating the variant located within ±2.5 kb of the mGene/eGene/Gene TSS were identified as CRE-promoter mQTLs/eQTLs/eQTMs while those located >2.5 kb from the mGene/eGene/Gene were identified as CRE-distal mQTLs/eQTLs/eQTMs. Number of unique mQTLs, eQTLs and eQTMs overlapping a SE indicating the variant located within ±2.5 kb of the mGene/eGene/Gene TSS were identified as SE-promoter mQTLs/eQTLs/eQTMs while those located >2.5 kb from the mGene/eGene/Gene were identified as SE-distal mQTLs/eQTLs/eQTMs b: Upper panel: LocusZoom plots of *EML1* eQTLs (genotype and *EML1* expression association) and CpG cg26180015 mQTLs (genotype and cg26180015 methylation association). The y axis shows

$-\log_{10}$(P value) of association tests from eQTLs and mQTLs. Points are color coded based on linkage disequilibrium (LD) (r^2) relative to the highlighted variant rs11625037 identified as the most significant in E2M_SMR analysis of eQTLs and mQTLs and M2E_SMR analysis of mQTLs and eQTLs. The lower panel includes associations identified in E2M_SMR and M2E_SMR for *EML1* gene. Tracks represent the chromatin loops, E2M_SMR variant, M2E_SMR variant, SEs, CREs, H3K27Ac coverage, genes, and Hi-C physical contact maps identifying TADs. c: Upper panel: LocusZoom plots of *LPIN1* eQTLs (genotype and *LPIN1* expression association) and CpG cg25654774 mQTLs (genotype and cg25654774 methylation association). The y axis shows $-\log_{10}$(P value) of association tests from eQTLs and mQTLs. Points are color coded based on linkage disequilibrium (LD) (r^2) relative to the highlighted variant rs3795974 identified as the most significant in EM analysis of eQTLs and mQTLs using coloc. The lower panel includes associations identified in EM for *LPIN1* gene. Tracks represent the chromatin loops, EM (colocalizing eQTL-mQTL) variant, SEs, CREs, H3K27Ac coverage, genes, and TADs. CRE, *Cis*-regulatory element. SE, Super-enhancer. TADs, Topologically associating domain.
