# Supplementary material for: QTL mapping of human retina DNA methylation identifies 87 gene-epigenome interactions in age-related macular degeneration
Source: Nat Commun. 2024 Mar 4;15:1972. doi: 10.1038/s41467-024-46063-8 (PMC10912779; doi:10.1038/s41467-024-46063-8)
Supplement: Supplementary file 3 — Description of Additional Supplementary Files [file 41467_2024_46063_MOESM3_ESM.pdf]

Description of Additional Supplementary Files

Supplementary Data 1: List of retina donors used for DNA methylation (n=160).

Supplementary Data 2: List of independent retina mQTLs and CpGs
Description: This table contains 37,453 independent mQTL signals identified with QTLtools using conditional analysis (n=152). Empirical p-values were calculated for each CpG by using permutation analysis and fitting a beta distribution on the genotype regression coefficient p-values of all variants tested per CpG. Storey & Tibshirani False Discovery Rate (FDR) was applied to the empirical mQTL p-values to correct for the total number of CpGs tested. Significance was determined at FDR≤0.05. The summary statistics (nominal p-values, beta and standard error) for all significant variant-CpG pair mQTLs can be downloaded from Zenodo at: https://doi.org/10.5281/zenodo.10569726.

Supplementary Data 3: List of independent retina eQTLs and eGenes
Description: This table contains 12,505 independent eQTL signals identified with QTLtools using conditional analysis (n=403). Empirical P-values were calculated per eGene using beta-distribution fit with permutations on the genotype regression coefficient p-values of all variants tested per gene. Storey & Tibshirani False Discovery Rate (FDR) was applied to the empirical eQTL p-values to correct for the total number of genes tested. Significance was determined at FDR≤0.05. The summary statistics (nominal p-values, beta and standard error) for all significant variant-gene pair eQTLs can be downloaded from Zenodo at: https://doi.org/10.5281/zenodo.10569726.

Supplementary Data 4: Summary of gene ontology enrichment results for retina mGenes using GeneEnrich
Description: This table contains gene set enrichment analysis results for retina mGenes. The target genes of mQTLs with FDR≤0.05 (mGenes, genes mapped to target CpGs) were tested for enrichment in predefined gene sets using GeneEnrich, a hypergeometric-based test (one-sided). Gene Ontology (GO), Reactome, and Kyoto Encyclopedia of Genes and Genomes (KEGG) gene sets were tested, considering only the subset of genes present in the given database, tested for mQTLs, and expressed in retina. Enrichment results are shown for gene sets that passed Benjamini-Hochberg false discovery rate FDR<0.05.

Supplementary Data 5: Summary of gene ontology enrichment results for retina eGenes using GeneEnrich
Description: This table contains gene set enrichment analysis results for eGenes. The target genes of eQTLs (eGenes) with FDR≤0.05 were tested for enrichment in predefined gene sets using GeneEnrich, a hypergeometric-based test (one-sided). Gene Ontology (GO), Reactome, and Kyoto Encyclopedia of Genes and Genomes (KEGG) gene sets were tested and only the subset of genes in each gene set found in the given database, expressed in retina and tested for eQTLs were considered in the analysis. Enrichment results are shown for gene sets that passed Benjamini-Hochberg false discovery rate FDR<0.05.

Supplementary Data 6: List of mCpGs and their tissue-specificty across five tissues, and list of retina-specific mQTLs
Description: Retina mCpGs were compared with CpGs found in adipose, blood, endometrium, frontal cortex, and skeletal muscle tissues. Results are shown for mCpGs shared in 1-5 tissues and mCpGs specific to retina. Retina mQTLs were compared with mQTLs from adipose, blood, endometrium, frontal cortex and skeletal muscle tissues. The second sheet lists mQTLs specific to retina with their corresponding p-values.

Supplementary Data 7: Summary of gene ontology enrichment results of retina-specific mQTL target genes using GeneEnrich
Description: This table contains gene set enrichment analysis results for retina-specific mQTL target genes (mGenes), using GeneEnrich, a hypergeometric-based test (one-sided). Gene Ontology (GO), Reactome, and Kyoto Encyclopedia of Genes and Genomes (KEGG) gene sets were tested, considering only the subset of genes in each gene set found in the given database that are expressed in retina and that map to CpGs tested for mQTLs. Enrichment results are shown for gene sets that passed Benjamini-Hochberg false discovery rate FDR<0.05.

Supplementary Data 8: List of independent retina eQTMs
Description: This table contains all independent retina eQTMs (n=152) identified using a linear regression model implemented in R by testing associations between methylation of CpG sites and expression levels of genes whose TSS is within ±1Mb of each CpG site. P-values were calculated for the CpG regression coefficient, and empirical p-values for the eQTM target genes were estimated using permutation analysis and beta distribution fit on the p-values of all CpGs tested per gene. Benjamini-Hochberg (BH) False Discovery Rate (FDR) procedure was applied to the eQTM empirical p-values to account for the number of genes tested. Significant eQTMs were determined at FDR≤0.05. Pearson's correlation coefficient was calculated for M-value and gene expression value for all significant eQTMs. The summary statistics (nominal p-values, beta and standard error) for all significant CpG-gene pair eQTMs can be downloaded from Zenodo at: https://doi.org/10.5281/zenodo.10569726.

Supplementary Data 9: Summary of gene ontology and pathway enrichment results for eQTM target genes using GeneEnrich
Description: This table contains gene set enrichment analysis results for eQTM target genes (FDR≤0.05) using GeneEnrich, a hypergeometric-based test (one-sided). Gene Ontology (GO), Reactome, and Kyoto Encyclopedia of Genes and Genomes (KEGG) gene sets were tested, considering only the subset of eQTM target genes in each gene set found in the given database, expressed in retina and tested for eQTMs. Enrichment results are shown for gene sets that passed Benjamini-Hochberg false discovery rate FDR<0.05.

Supplementary Data 10: Summary of gene set enrichment results for sets of more than 10 eQTM target genes regulated by the same CpG using GeneEnrich
Description: This table contains gene set enrichment results for sets of 10 or more target genes regulated by the same CpG, using GeneEnrich, a hypergeometric-based test (one-sided). Gene Ontology (GO), Reactome, Kyoto Encyclopedia of Genes and Genomes (KEGG), and mouse phenotype ontology from the Mouse Genome Informatics (MGI) gene sets were tested, considering only the subset of genes in each gene set found in the given database, expressed in

retina and tested for eQTMs. Enrichment results are shown for gene sets that passed Benjamini-Hochberg false discovery rate FDR<0.05.

Supplementary Data 11: Summary of gene set enrichment results of eQTM target genes on chr16 using GeneEnrich
Description: This table contains gene set enrichment results for eQTM target genes for chr16, using GeneEnrich, a hypergeometric-based test (one-sided). Gene Ontology (GO), Reactome, and Kyoto Encyclopedia of Genes and Genomes (KEGG) gene sets were tested, considering only the subset of genes in each gene set found in the given database, expressed in retina and tested for eQTMs. Enrichment results are shown for gene sets that passed Benjamini-Hochberg false discovery rate FDR<0.05.

Supplementary Data 12: Summary of gene set enrichment results of eQTM target genes on chr19 using GeneEnrich
Description: This table contains gene set enrichment results for eQTM target genes on chr19, using GeneEnrich, a hypergeometric-based test (one-sided). Gene Ontology (GO), Reactome, and Kyoto Encyclopedia of Genes and Genomes (KEGG) gene sets were tested, considering only the subset of genes in each gene set found in the given database, expressed in retina and tested for eQTMs. Enrichment results are shown for gene sets that passed Benjamini-Hochberg false discovery rate FDR<0.05.

Supplementary Data 13: Summary data-based mendelian randomization (SMR) results for the effect of eQTLs on mQTLs in retina (E2M_SMR)
Description: This table contains all the significant E2M_SMR associations (considering gene expression as the exposure and DNA methylation as the outcome) identified with summary data-based mendelian randomization (SMR) analysis, using the GSMR (v1.1.1) package. Heterogeneity independent instruments (HEIDI) test and P-value threshold of greater than 0.05 were used to differentiate pleiotropy from linkage. Bonferroni correction was used to determine significance at $\alpha<0.05$.

Supplementary Data 14: Summary data-based mendelian randomization (SMR) results for the effect on mQTLs on eQTLs in retina (M2E_SMR)
Description: This table contains all the significant M2E_SMR associations (considering DNA methylation as the exposure and gene expression as the outcome) identified with summary data-based mendelian randomization (SMR) analysis, using the GSMR (v1.1.1) package. Heterogeneity independent instruments (HEIDI) test and P-value threshold of greater than 0.05 were used to differentiate pleiotropy from linkage. Bonferroni correction was used to determine significance at $\alpha<0.05$.

Supplementary Data 15: Summary of gene ontology and pathway enrichment results of E2M_SMR and M2E_SMR common genes using GeneEnrich
Description: This table contains gene set enrichment results for common genes identified in both the E2M_SMR and M2E_SMR analyses that identified causal or pleiotropic associations between eQTLs and mQTLs (E2M_SMR) and vice versa (M2E_SMR), using GeneEnrich, a hypergeometric-based test (one-sided). Gene Ontology (GO), Reactome, and Kyoto Encyclopedia of Genes and Genomes (KEGG) gene sets were tested, considering only the subset of genes in

each gene set found in the given database, expressed in retina, and identified in the SMR analysis. Enrichment results are shown for gene sets that passed Benjamini-Hochberg false discovery rate FDR<0.05. SMR, Summary data-based mendelian randomization.

Supplementary Data 16: Summary of colocalization results using coloc between retina eQTLs and mQTLs (EM)
Description: This table contains the significant colocalization results identified between retina eQTLs and mQTLs, using coloc. Coloc analysis was performed to identify shared causal variants between mQTL and eQTL. Significance was determined based on posterior probability (PPA) >0.8.

Supplementary Data 17: Summary of gene set enrichment results for colocalizing retina eQTLs and mQTLs (EM) based on coloc using GeneEnrich
Description: This table contains gene set enrichment analysis (GSEA) results for genes identified in the eQTL and mQTL (EM) colocalization analysis using coloc. GeneEnrich, a hypergeometric-based test (one-sided) was used to perform the GSEA. Gene Ontology (GO), Reactome, and Kyoto Encyclopedia of Genes and Genomes (KEGG) gene sets were tested, considering only the subset of genes in each gene set found in the given database, expressed in retina, and identified in coloc analysis. Enrichment results are shown for gene sets that passed Benjamini-Hochberg false discovery rate FDR<0.05.

Supplementary Data 18: Summary of associations identified in SMR analysis of mQTLs with AMD GWAS
Description: This table contains all the significant associations identified in the Summary data-based mendelian randomization (SMR) analysis of retina mQTLs with AMD GWAS. SMR analysis was used to test for causal or pleiotropic associations between mQTLs and AMD GWAS using the GSMR (v1.1.1) package. Heterogeneity independent instruments (HEIDI) test and P-value threshold of greater than 0.05 were used to differentiate pleiotropy from linkage. Bonferroni correction was used to determine significance at $\alpha<0.05$.

Supplementary Data 19: Summary of associations identified in SMR analysis of eQTLs with AMD GWAS
Description: This table contains all the significant associations identified in the Summary data-based mendelian randomization (SMR) analysis of eQTLs with AMD GWAS. SMR analysis was used to test for causal or pleiotropic associations between eQTL and AMD GWAS using the GSMR (v1.1.1) package. Heterogeneity independent instruments (HEIDI) test and P-value threshold of greater than 0.05 were used to differentiate pleiotropy from linkage. Bonferroni correction was used to determine significance at $\alpha<0.05$.

Supplementary Data 20: Summary of colocalization results identified in eCAVIAR analysis of mQTL and/or eQTLs with AMD GWAS loci
Description: This table contains the significant eCAVIAR colocalization results for 52 independent AMD GWAS variants at 34 loci by QTL type and target gene combinations tested for all overlapping retina mQTLs and retina eQTLs. Significance was determined at a colocalization posterior probability (CLPP) > 0.01. To filter out potential false positives, we flagged cases where the GWAS p-value of the colocalization eVariant or mVariant was above 0.01 or whose e/mQTL

p-values was above 10^{-4} and/or did not pass FDR below 0.05 (FALSE in Column: 'Pass_QC_QTL_FDR05_P1E04_GWAS_P1E05'). All e/mVariants within an LD interval (r^2>0.1 plus 50kb on either side) around each lead AMD GWAS variant were tested per locus. The gene symbol for the eQTL refers to eQTL target gene (eGene) and for the mQTL refers to the potential mQTL target (mGene) defined using Illumina's annotation, which is based on the UCSC RefGene mapping of CpGs to genes. The second sheet is a summary of the eCAVIAR colocalization results for each AMD GWAS locus that had at least one significantly colocalizing retina mQTL and/or eQTL (CLPP>0.01). The direction of effect of each colocalizing e/mQTL relative to AMD risk is given as '+' or '-' in square brackets. The number preceding the +/- signs refers to the number of tissues in which the eQTL (e) or mQTL (m) has the given direction of effect on AMD risk.

Supplementary Data 21: Summary of colocalization results between AMD GWAS (GM) and mQTLs using coloc
Description: This table contains the significant colocalization results using coloc identified between AMD GWAS and mQTL (GM). coloc analysis was performed to estimate the posterior probability that a lead variant is associated with AMD GWAS and mQTL. Significance was determined based on posterior probability (PPA) >0.8, providing evidence that the two associations share the same causal variant.

Supplementary Data 22: Summary of colocalization results between AMD GWAS (GE) and eQTLs using coloc
Description: This table contains the significant colocalization results using coloc identified between AMD GWAS and eQTL (GE). coloc analysis was performed to estimate the posterior probability that a lead variant is associated with AMD GWAS and eQTL. Significance was determined based on posterior probability (PPA) >0.8, providing evidence that the two associations share the same causal variant.

Supplementary Data 23: List of common colocalization results between the AMD GWAS and mQTLs (GM) or eQTLs (GE) based on coloc
Description: This table contains the common colocalization results identified between AMD GWAS versus mQTLs (GM) and AMD GWAS versus eQTLs (GE) using coloc.

Supplementary Data 24: Summary of colocalization results using moloc comparing AMD GWAS with retina eQTLs and mQTLs (GEM)
Description: This table contains the significant moloc colocalization results identified between AMD GWAS, retina eQTL (E) and retina mQTL (M). Colocalization analysis was performed with moloc to estimate the posterior probability that a given variant is associated with AMD GWAS, retina eQTL and retina mQTL. Significance was determined based on posterior probability (PPA) >0.8, providing evidence that three associations share the same causal variant.

Supplementary Data 25: List of common CpGs and genes identified in SMR analysis (E2M_SMR, M2E_SMR) and coloc analysis of retina eQTLs and mQTLs (EM)
Description: This table contains the list of common CpGs and genes identified with colocalization analysis of eQTLs and mQTLs (EM) using coloc, and Summary data-based mendelian randomization (SMR) analysis of E2M_SMR, considering eQTLs as the exposure and mQTL as the outcome, and of M2E_SMR, considering mQTLs as the exposure and eQTLs as the outcome.

Supplementary Data 26: List of retina mQTLs, eQTLs and eQTMs and their associated compartment from Hi-C data in retina

Description: This table contains three sheets with lists of retina mQTLs, eQTLs and eQTMs specifying their overlap with chromatin compartment A or B, based on analysis in R of retina Hi-C data from Marchal *et al.*, Nature Communications 2022 (PMID: 36207300).

Supplementary Data 27: List of retinal mQTLs and their associated features with Hi-C loops in retina

Description: This table contains a list of mQTLs whose mVariants and their target or non-target genes overlap with Hi-C loop foot locations in retina (Marchal *et al.*, Nature Communications 2022; PMID: 36207300). mQTL variants located within ±2.5 kb of the mGene transcription start site (TSS) were defined as promoter mQTLs and those located >2.5 kb from the mGene were defined as distal mQTLs. mVariants in contact with a promoter or distal mGene other than the target mGene were defined as promoter or distal non-mGene mQTLs, respectively. All overlaps were performed using GenomicRanges in R.

Supplementary Data 28: List of retinal eQTLs and their associated features with Hi-C loops in retina

Description: This table contains a list of eQTLs whose eVariants and their target or non-target genes overlap with Hi-C loop foot locations in retina (Marchal *et al.*, Nature Communications 2022; PMID: 36207300). eQTL variants located within ±2.5 kb of the eGene transcription start site (TSS) were defined as promoter eQTLs and those located >2.5 kb from the eGene were defined as distal eQTLs. eVariants in contact with a promoter or distal eGene other than the target eGene were defined as promoter or distal non-eGene eQTLs, respectively. All overlaps were performed using GenomicRanges in R.

Supplementary Data 29: List of retinal eQTMs and their associated features with Hi-C loops in retina

Description: This table contains list of eQTMs whose CpGs and their target genes overlap with a Hi-C loop foot locations in retina (Marchal *et al.*, Nature Communications 2022; PMID: 36207300). eQTM CpGs which overlap a chromatin loop foot were classified based on the location contacted by opposite loop foot; CpG in contact with their target gene promoter (within ±2.5 kb of TSS) were defined as Gene promoter eQTMs, and CpGs in contact with a promoter other than the target gene were defined as non-Gene promoter eQTMs. CpGs located >2.5 kb from the target gene were defined as distal eQTMs. All overlaps were performed using GenomicRanges in R.

Supplementary Data 30: List of retinal mQTLs and their associated features with retinal CREs and SEs

Description: This table contains a list of mQTLs whose mVariants and target genes (mGenes) overlap *cis*-regulatory element (CREs) and super-enhancers (SEs) identified from epigenetic data in retina (Marchal *et al.*, Nature Communications 2022; PMID: 36207300). All overlaps were performed using GenomicRanges (v1.42) in R. mQTL variants located within ±2.5 kb of the mGene transcription start site (TSS) were defined as promoter mQTLs and those located >2.5 kb from the mGene were defined as distal mQTLs. mVariants in contact with a promoter of or distal

to an mGene other than the target mGene were defined as promoter or distal non-mGene mQTLs, respectively.

Supplementary Data 31: List of retinal eQTLs and their associated features with retinal CREs and SEs
Description: This table contains a list of eQTLs whose eVariants and target genes (eGenes) overlap *cis*-regulatory element (CREs) and super-enhancers (SEs) identified from epigenetic data in retina (Marchal *et al*., Nature Communications 2022; PMID: 36207300). All overlaps were performed using GenomicRanges (v1.42) in R. eQTL variants located within ±2.5 kb of the eGene transcription start site (TSS) were defined as promoter eQTLs and those located >2.5 kb from the eGene were defined as distal eQTLs. eVariants in contact with a promoter of or distal to an eGene other than the target eGene were defined as promoter or distal non-eGene eQTLs, respectively.

Supplementary Data 32: List of retinal eQTMs and their associated features with retinal CREs and SEs
Description: This table contains a list of eQTMs whose CpGs and target genes overlap *cis*-regulatory element (CREs) and super-enhancers (SEs) identified from epigenetic data in retina (Marchal *et al*., Nature Communications 2022; PMID: 36207300). All overlaps were performed using GenomicRanges (v1.42) in R. eQTMs whose CpGs are within ±2.5 kb of their target gene TSS were defined as promoter eQTMs and those located >2.5 kb from the target gene were defined as distal eQTMs. eQTMs whose CpGs are in contact with a promoter of or distal to a gene other than the target gene were defined as promoter or distal non-target gene eQTMs, respectively.

Supplementary Data 33: List of associations identified in SMR analysis of mQTL with AMD GWAS and the linked target genes with retinal Hi-C loops, CREs and SEs
Description: This table contains associations identified by Summary data-based mendelian randomization (SMR) analysis of retina mQTLs with AMD GWAS for which there is support for the link with the target genes by retinal Hi-C loops, *cis*-regulatory element (CREs), and super-enhancers (SEs) (taken from Marchal *et al*., Nature Communications 2022; PMID: 36207300). To confirm target genes for associations identified in SMR, mVariants and CpGs identified in these associations were overlapped with Hi-C loops, CRE, and SE. For a loop target gene, one foot of the loop overlaps the variant, and the second foot of the loop overlaps the gene body or TSS of a gene. CRE and SE target genes were defined by both the mVariant and gene body (or TSS of a gene) overlapping the same CRE or SE using bedtools.

Supplementary Data 34: List of associations identified in SMR analysis of eQTL with AMD GWAS and the linked target genes with retinal Hi-C loops, CREs and SEs
Description: This table contains associations identified by Summary data-based mendelian randomization (SMR) analysis of retina eQTLs with AMD GWAS for which there is support for the link with the target genes by retinal Hi-C loops, *cis*-regulatory element (CREs), and super-enhancers (SEs) (taken from Marchal *et al*., Nature Communications 2022; PMID: 36207300). To confirm target genes for associations identified in SMR, eVariants and target genes identified in these associations were overlapped with Hi-C loops, CRE, and SE. For a loop target gene, one foot of the loop overlaps the variant, and the second foot of the loop overlaps the gene body or TSS of a gene. CRE and SE target genes were defined by both the eVariant and gene body (or TSS of a gene) overlapping the same CRE or SE using bedtools.

Supplementary Data 35: List of colocalizations identified in eCAVIAR analysis of mQTL with AMD GWAS and the linked target genes with retinal Hi-C loops and CREs

Description: This table contains associations identified by eCAVIAR colocalization analysis between retina mQTL and AMD GWAS loci, supported by overlap with retinal Hi-C loops, *cis*-regulatory element (CREs), and super-enhancers (SEs) (taken from Marchal *et al*., Nature Communications 2022; PMID: 36207300). For a loop target gene, one foot of the loop had to overlap an mVariant, and the second foot of the loop had to overlap the gene body or TSS of the gene mapped to the associated CpG. CRE and SE target genes were defined by mVariant and gene body (or TSS of a gene) overlapping the same CRE or SE using bedtools. Only overlap with CRE was found.

Supplementary Data 36: List of associations identified in SMR analysis of E2M_SMR and the linked target genes with retinal Hi-C loops, CREs and SEs

Description: This table contains associations identified by Summary data-based mendelian randomization (SMR) analysis of E2M_SMR (eQTLs treated as the exposure and mQTLs as the outcome), where support was found for the target genes by Hi-C loops, *cis*-regulatory element (CREs), and super-enhancers (SEs) (Marchal *et al*., Nature Communications 2022; PMID: 36207300). To confirm target genes for associations identified in E2M_SMR, variants identified in these associations were overlapped with Hi-C loops, CRE, and SE. For a loop target gene, one foot of the loop had to overlap the variant, and the second foot of the loop had to overlap the gene body or TSS of the gene. CRE and SE target genes were defined by variant and the gene body (or TSS of gene) overlapping the same CRE or SE using bedtools.

Supplementary Data 37: List of associations identified in SMR analysis of M2E_SMR and the linked target genes with retinal Hi-C loops, CREs and SEs

Description: This table contains associations identified by Summary data-based mendelian randomization (SMR) analysis of M2E_SMR (mQTLs treated as the exposure and eQTLs as the outcome), where support was found for the target genes by Hi-C loops, *cis*-regulatory element (CREs), and super-enhancers (SEs) (Marchal *et al*., Nature Communications 2022; PMID: 36207300). To confirm target genes for associations identified in M2E_SMR, variants identified in these associations were overlapped with Hi-C loops, CRE, and SE. For a loop target gene, one foot of the loop had to overlap the variant, and the second foot of the loop had to overlap the gene body or TSS of the gene. CRE and SE target genes were defined by variant and the gene body (or TSS of gene) overlapping the same CRE or SE using bedtools.

Supplementary Data 38: List of retinal eQTL-mQTL colocalizations identified with coloc and the linked target genes supported with retinal Hi-C loops, CREs and SEs

Description: This table contains colocalizations results identified by coloc analysis of retinal eQTL-mQTL (EM) with support for associated target genes by retinal Hi-C loops, *cis*-regulatory element (CREs), and super-enhancers (SEs) (Marchal *et al*., Nature Communications 2022; PMID: 36207300). To confirm target genes for associations identified in the coloc analysis, variants identified in these associations were overlapped with loops, CRE, and SE. For a loop target gene, one foot of the loop overlaps the variant, and the second foot of the loop overlaps the gene body or TSS of the gene. CRE and SE target genes were defined by the variant and gene body (or TSS of a gene) overlapping the same CRE or SE using bedtools.

Supplementary Data 39: List of non-redundant target genes identified in retinal mQTL and AMD GWAS analyses using SMR, eCAVIAR, coloc and moloc.

Description: This table list the 87 genes proposed to affect age-related macular degeneration (AMD) risk through DNA methylation and/or gene-expression changes based on Summary data-based Mendelian randomization (SMR) analysis and colocalization analyses of retina mQTLs and AMD GWAS with eCAVIAR and coloc, and between retina mQTLs, eQTLs, and AMD GWAS with moloc. Cases where no genes are listed in the 'Locus' column refer to newly proposed AMD associations based on the MR or colocalization analyses.
