# Supplementary material for: QTL mapping of human retina DNA methylation identifies 87 gene-epigenome interactions in age-related macular degeneration
Source: Nat Commun. 2024 Mar 4;15:1972. doi: 10.1038/s41467-024-46063-8 (PMC10912779; doi:10.1038/s41467-024-46063-8)
Supplement: Supplementary file 43 — Reporting Summary [file 41467_2024_46063_MOESM43_ESM.pdf]

nature portfolio

Corresponding author(s): Anand Swaroop, Ayellet V. Segrè

Last updated by author(s): Jan 25, 2024

Reporting Summary

Nature Portfolio wishes to improve the reproducibility of the work that we publish. This form provides structure for consistency and transparency in reporting. For further information on Nature Portfolio policies, see our Editorial Policies and the Editorial Policy Checklist.

Statistics

For all statistical analyses, confirm that the following items are present in the figure legend, table legend, main text, or Methods section.

n/a	Confirmed	
☐	☒	The exact sample size (*n*) for each experimental group/condition, given as a discrete number and unit of measurement
☐	☒	A statement on whether measurements were taken from distinct samples or whether the same sample was measured repeatedly
☐	☒	The statistical test(s) used AND whether they are one- or two-sided<br>*Only common tests should be described solely by name; describe more complex techniques in the Methods section.*
☐	☒	A description of all covariates tested
☐	☒	A description of any assumptions or corrections, such as tests of normality and adjustment for multiple comparisons
☐	☒	A full description of the statistical parameters including central tendency (e.g. means) or other basic estimates (e.g. regression coefficient) AND variation (e.g. standard deviation) or associated estimates of uncertainty (e.g. confidence intervals)
☐	☒	For null hypothesis testing, the test statistic (e.g. *F*, *t*, *r*) with confidence intervals, effect sizes, degrees of freedom and *P* value noted<br>*Give P values as exact values whenever suitable.*
☐	☒	For Bayesian analysis, information on the choice of priors and Markov chain Monte Carlo settings
☐	☒	For hierarchical and complex designs, identification of the appropriate level for tests and full reporting of outcomes
☐	☒	Estimates of effect sizes (e.g. Cohen's *d*, Pearson's *r*), indicating how they were calculated

Our web collection on statistics for biologists contains articles on many of the points above.

Software and code

Policy information about availability of computer code

Data collection	No software was used in the collection of data in this study.
Data analysis	Publicly available software and packages were used throughout this study according to each developer's instructions. Custom scripts generated for the study will be available upon request. Data analysis in this paper was performed using the following software: Eigenstrat v6.1.4 (https://github.com/DReichLab/EIG), minfi v1.42.0 (https://bioconductor.org/packages/release/bioc/html/minfi.html), sva v3.38.0 (https://bioconductor.org/packages/release/bioc/html/sva.html), limma v3.46.0 (https://bioconductor.org/packages/release/bioc/html/limma.html), QTLtools v1.3.1 (https://qtltools.github.io/qtltools/), edgeR v3.32.1 (https://bioconductor.org/packages/release/bioc/html/edgeR.html), TORUS no version number provided (https://github.com/xqwen/torus), Ensembl's Variant Effect Predictor (VEP) and Loss-Of-Function Transcript Effect Estimator (LOFTEE) v100 (https://github.com/Ensembl/ensembl-vep), BCFtools v1.11 (http://www.htslib.org/download/), FastQTL no version number provided (https://github.com/francois-a/fastqtl), ggforestplot v0.1.0 (https://github.com/NightingaleHealth/ggforestplot), GeneEnrich (https://github.com/segrelabgenomics/GeneEnrich), GSMR v1.1.1 (https://yanglab.westlake.edu.cn/software/gsmr/#Sourcecode), eCAVIAR (https://github.com/fhormoz/caviar), coloc v5.1.1 (https://github.com/chr1swallace/coloc), moloc v0.10 (https://github.com/clagiamba/moloc), GenomicRanges v1.42 (https://bioconductor.org/packages/release/bioc/html/GenomicRanges.html), bedtools v2.27.1 (https://github.com/arq5x/bedtools2), IGV v2.15.2 (https://software.broadinstitute.org/software/igv/download), LocusZoom v1.4 (http://locuszoom.org/), LocusComparer v1.0.0 (https://github.com/boxiangliu/locuscomparer), GO-Figure v1.0.1 (https://gitlab.com/evogenlab/GO-Figure), IGV v2.15.2 (https://software.broadinstitute.org/software/igv/download), PLINK v1.90 (https://www.cog-genomics.org/plink2/) R v3.5.0 and v4.0.0 (https://cran.r-project.org/), Washington University Epigenome browser: http://epigenomegateway.wustl.edu/browser/, qctool v2 (https://www.well.ox.ac.uk/~gav/qctool_v2/).

For manuscripts utilizing custom algorithms or software that are central to the research but not yet described in published literature, software must be made available to editors and reviewers. We strongly encourage code deposition in a community repository (e.g. GitHub). See the Nature Portfolio guidelines for submitting code & software for further information.

Data

Policy information about availability of data

All manuscripts must include a data availability statement. This statement should provide the following information, where applicable:

- Accession codes, unique identifiers, or web links for publicly available datasets
- A description of any restrictions on data availability
- For clinical datasets or third party data, please ensure that the statement adheres to our policy

The data that support this study are available from the corresponding authors upon reasonable request. Dataset produced in this study are accessible in GEO under the accession number: GSE231536 [https://www.ncbi.nlm.nih.gov/geo/query/acc.cgi?acc=GSE231536] (Methylation array). Gene expression data are from our previous study, under the accession GSE115828 [https://www.ncbi.nlm.nih.gov/geo/query/acc.cgi?acc=GSE115828]. The summary statistics (nominal p-values, beta and standard error) for all significant variant-CpG pair mQTLs, all significant variant-gene pair eQTLs, and all significant CpG-gene pair eQTMs are provided on Zenodo at the following URL: https://doi.org/10.5281/zenodo.10569726. Gene Ontology (GO) (http://geneontology.org/), Reactome, and Kyoto Encyclopedia of Genes and Genomes (KEGG) gene sets were downloaded from the Molecular Signature Database (MSigDB) (https://www.gsea-msigdb.org/gsea/msigdb/). The GWAS summary statistics for AMD are accessible under accession GCST00321926 [https://www.ebi.ac.uk/gwas/studies/GCST003219].

Research involving human participants, their data, or biological material

Policy information about studies with human participants or human data. See also policy information about sex, gender (identity/presentation), and sexual orientation and race, ethnicity and racism.

Reporting on sex and gender	We used 160 deidentified postmortem human retina samples for DNA methylation and mQTL analyses with equal distribution of males and females. Details about the sex of samples are provided in Supplementary Table 1. Sex was used as covariate in the SVA analysis.
Reporting on race, ethnicity, or other socially relevant groupings	We used 160 deidentified postmortem human retina samples for DNA methylation and mQTL analyses. All the samples belong to European ancestry.
Population characteristics	We used 160 deidentified postmortem human retina samples for DNA methylation and mQTL analyses with equal distribution of males and females and mean age of 73 years. Additional details about the samples are provided in Supplementary Table 1.
Recruitment	Post-mortem deidentified human donor eyes were procured by the Minnesota Lions Eye Bank after informed consent from the donor or next of kin and in accordance with the tenets of the Declaration of Helsinki. The unidentified retina samples were obtained for DNA methylation. Human retina tissue is difficult to obtain. We obtained small punches of postmortem retina from controls and cases from University of Minnesota (Dr. Deb Ferrington). These samples were converted into DNA. Almost all of the DNA has been used for obtaining the methylation data.
Ethics oversight	For the Bethesda dataset, postmortem peripheral retina samples from 96 deidentified donors were procured by the Minnesota Lions Eye Bank in accordance with the tenets of the Declaration of Helsinki and following informed consent from the donors or their family. Given that deidentified post-mortem samples were used, the study was exempted by the institutional review boards of the University of Minnesota and National Eye Institute, National Institutes of Health.<br>For the Cologne and Munich dataset, 64 peripheral retina samples were collected at the Ludwig-Maximilians-University (LMU) Munich and the University of Cologne Eye Bank after informed consent from the donor or next of kin was obtained. This was done in full accordance with the tenets of the Declaration of Helsinki. The tissue and data collections and the subsequent study was approved by the local Ethics Boards at the LMU (Application nr. MUC73416) and the University of Cologne (application nr. 14-247), respectively.

Note that full information on the approval of the study protocol must also be provided in the manuscript.

Field-specific reporting

Please select the one below that is the best fit for your research. If you are not sure, read the appropriate sections before making your selection.

☒ Life sciences ☐ Behavioural & social sciences ☐ Ecological, evolutionary & environmental sciences

For a reference copy of the document with all sections, see nature.com/documents/nr-reporting-summary-flat.pdf

Life sciences study design

All studies must disclose on these points even when the disclosure is negative.

Sample size	We used 160 deidentified postmortem human retina samples for DNA methylation as these samples were collected from 2 different locations USA and Germany for the mQTL analyses. Previous QTL studies have shown that this size is sufficient to detect eQTLs and mQTLs (PMID:32913098).
Data exclusions	No data were excluded from these analyses.

Replication	We replicated mQTLs identified in five different tissues (Adipose, blood, Endometrium, Frontal cortex and Skeletal muscle) in our data based on the availability of mQTL datasets when the project started. This is a partial replication to validate our mQTLs and identify tissue-specific mQTLs.
Randomization	We randomized AMD cases and controls in our retina samples to make sure that they were distributed evenly across DNA isolation dates.
Blinding	We did not perform any blinding. The information on post-mortem retina samples was essential for the analysis. We needed to know AMD status of samples before performing analysis in order to correct for disease status in the QTL analysis.

Reporting for specific materials, systems and methods

We require information from authors about some types of materials, experimental systems and methods used in many studies. Here, indicate whether each material, system or method listed is relevant to your study. If you are not sure if a list item applies to your research, read the appropriate section before selecting a response.

Materials & experimental systems

n/a	Involved in the study
☒	☐ Antibodies
☒	☐ Eukaryotic cell lines
☒	☐ Palaeontology and archaeology
☒	☐ Animals and other organisms
☒	☐ Clinical data
☒	☐ Dual use research of concern
☒	☐ Plants

Methods

n/a	Involved in the study
☒	☐ ChIP-seq
☒	☐ Flow cytometry
☒	☐ MRI-based neuroimaging
